# Supplementary material for: MetaQTL: a package of new computational methods for the meta-analysis of QTL mapping experiments
Source: BMC Bioinformatics. 2007 Feb 8;8:49. doi: 10.1186/1471-2105-8-49 (PMC1808479; doi:10.1186/1471-2105-8-49)
Supplement: Additional File 7 — MetaQTL Package : jar file and tutorial. This Zip archive contains both the MetaQTL JAR file and the files of the tutorial. [file 1471-2105-8-49-S7.zip › org.inra.metaqtl/doc/org/metaqtl/algo/package-frame.html]

org.metaqtl.algo


org.metaqtl.algo

Classes 
  
ClustAlgorithm
  
CMSAlgorithm
  
EMAlgorithm
  
HClustAlgorithm
  
MetaAlgorithm
  
MetaMapAlgorithm
  
QtlClustAlgorithm
  
QtlProjAlgorithm
  
QtlTreeAlgorithm
  
SVDAlgorithm
